# Soft Materials for Wearable/Flexible Electrochemical Energy Conversion, Storage, and Biosensor Devices

**DOI:** 10.3390/ma13122733

**Published:** 2020-06-16

**Authors:** Patrizia Bocchetta, Domenico Frattini, Srabanti Ghosh, Allibai Mohanan Vinu Mohan, Yogesh Kumar, Yongchai Kwon

**Affiliations:** 1 Dipartimento di Ingegneria dell’Innovazione, Università del Salento, via Monteroni, 73100 Lecce, Italy; 2 Graduate School of Energy and Environment, Seoul National University of Science and Technology, 232 Gongneung-ro, Nowon-gu, Seoul 01811, Korea; domenico.frattini@seoultech.ac.kr; 3 Department of Organic and Inorganic Chemistry, Universidad de Alcala (UAH), Alcalá de Henares, 28805 Madrid, Spain; srabanti.ghosh@uah.es; 4 Electrodics and Electrocatalysis Division, CSIR-Central Electrochemical Research Institute (CECRI), Karaikudi, Tamil Nadu 630003, India; vinumohan@cecri.res.in; 5 Department of Physics, ARSD College, University of Delhi, Delhi 110021, India; ykumar@arsd.du.ac.in; 6 Department of Chemical and Biomolecular Engineering, Seoul National University of Science and Technology, 232 Gongneung-ro, Nowon-gu, Seoul 01811, Korea

**Keywords:** soft materials, wearable devices, flexible polymer electrolyte fuel cells, enzymatic biofuel cells, embedded mediators, microbial fuel cells, flexible lithium batteries

## Abstract

Next-generation wearable technology needs portable flexible energy storage, conversion, and biosensor devices that can be worn on soft and curved surfaces. The conformal integration of these devices requires the use of soft, flexible, light materials, and substrates with similar mechanical properties as well as high performances. In this review, we have collected and discussed the remarkable research contributions of recent years, focusing the attention on the development and arrangement of soft and flexible materials (electrodes, electrolytes, substrates) that allowed traditional power sources and sensors to become viable and compatible with wearable electronics, preserving or improving their conventional performances.

## 1. Introduction

The advent of innovative flexible electronics facilitates the development of lightweight, portable and bendable devices like roll-up displays, intelligent mobile devices, electronic skin, implantable biomedical devices, and wearable biosensors [1,2,3,4,5,6,7,8]. Recently, some prototype flexible devices have emerged like organic light-emitting diode displays (LG), Youm flexible displays (Samsung), flexible smartphones (Philips), and so on. These smart devices necessitate the seamless integration of highly flexible energy storage or energy harvesting devices as a power source [9]. These flexible systems should possess appreciable mechanical resiliency to withstand the strain developed at harsh environmental conditions [10,11,12]. Furthermore, flexibility is a requisite parameter for challenging applications, such as space satellites [13], electric vehicles [14], and even buildings or other platforms with curvilinear surfaces [15,16].

The majority of the flexible devices rely on ultrathin plastic substrates like poly(ethylene terephthalate), poly(ethylene naphthalate), and polyimide, owing to their ink compatibility, bendability, mechanical and temperature stability [17]. For stretchable electronic skin applications, thin films of viscoelastic polymers like poly(dimethylsiloxane) (PDMS), polyurethane, and latex are the preferable substrates. For textile-based wearable applications, Gore-Tex fabric shows superior performance over other natural (cotton) and synthetic (polyester) fabrics, owing to its breathable and waterproof properties. The recent advances in nanostructured materials and ultrathin film fabrication technologies realize the development of highly flexible energy sources, of which supercapacitors, lithium-ion based batteries, and biofuel cells are typically leveraged [18]. The recent studies exploit conducting film networks of nanostructure materials, like zero-dimensional nanoparticles and nanospheres, one-dimensional nanotubes and nanowires, and three-dimensional nanoflakes and sheets, which are more susceptible to withstand vigorous mechanical deformations [19]. In addition, thin films facilitate fruitful heterogeneous integration, scalability, and large area compatibility. Various thin film-producing techniques, like solution processing, chemical, and physical vapor deposition, sputtering, and advanced lithographic patterning, were recently exploited for constructing mechanically resilient flexible power sources [20,21]. Additionally, extensive research is focused on developing nanoscopic electroactive materials, porous electrocatalysts, shape conformable electrodes, and strain sustaining structural configurations [22,23].

The incorporation of stretchable counterparts provides completely flexible systems that can mitigate the mechanical strain not only from bending agitations but also from other perturbations like compression, stretching, and twisting [24,25,26]. Stretchable electronics also extend their application towards long-lived bio-implantable polymer parts such as heart valves or stents [27]. Generally, the stretchable energy storage devices can be accomplished by depositing the electrically conductive active materials onto the pre-strained elastomeric substrate to form a wrinkled pattern [28]. Current research on flexible electronics is focused on implementing stretchability as a vital parameter for all energy storage or harvesting systems such as photovoltaic cells [29], supercapacitors [30], batteries [31], thermoelectric [32], piezoelectric [33] and triboelectric devices [34]. Yet, the excessive strain associated with these stretchable devices, beyond Young’s moduli, causes microscopic level cracks and damages. This problem can be addressed by introducing self-healing properties to the wearable electrodes and electrolytes which can autonomously cure the damages, and restore the electrical and ionic conductivities [35]. The intrinsic healing of the device by itself, without the support from external sources, instantaneously re-establishes energy storage or harvesting performances [36].

Conventional healthcare monitoring relies on centralized diagnosis at hospitals and time-consuming blood analysis by sophisticated complex instruments. This may obstruct patients in need of critical treatments and emergency care. The burgeoning wearable smart sensors offer an ideal platform for better healthcare or activity monitoring applications [37]. Such skin or textile-interfaced systems facilitate non-invasive sampling and analysis of body fluids like sweat, saliva, and tears, and provides timely diagnosis and precise therapy in a personalized manner [38,39,40]. Such wearable sensors also eliminate complex sample preparation and pre-treatment processes, and continuously monitor the dynamic level of relevant biomarkers. The desirable physical properties of the wearable devices to mount it on the skin include low elastic modulus and reversible response to large mechanical deformations up to ≈30% [41]. The wearable devices should facilitate robust bonding with the skin to minimize the stresses that could develop at the interface. The recent research is focused on developing a biocompatible, soft, skin-conformable platform for quantifying metabolites like lactate and glucose, enzymes, amino acids, and the electrolyte levels [42,43,44,45,46]. The conformal amalgamation of these devices onto the curvilinear and three-dimensional surfaces requires intrinsically flexible and stretchable active materials and substrates having similar mechanical properties [47,48,49,50,51,52]. The judicious integration of these flexible sensors and the energy storage devices are essential for better performance under the intense strain that developed during regular body motion and muscle movements. Biocompatibility is a vital parameter for all types of wearable devices. The compatibility issues subjected to direct attachment of flexible sensors onto the epidermis can be avoided by incorporating microfluidic channels that can facilitate real-time fluid transport to the sensor modalities.

In this paper, after a brief overview of wearable technologies, we report a survey of the most relevant literature works on soft materials for wearable/flexible fuel cells, batteries, supercapacitors, biofuel cells, and biosensors.

## 2. An Overview of Wearable Technologies

Large scale grid-connected energy harvesting and usage is not the only realistic energy-related application. Materials and methods for developing portable energy storage/conversion systems are inevitable for integrating with moving parts of equipment, robots, rugged field machines, vehicles, and non-planar surfaces. Stretchable energy storage devices that have elastic active components can be included in the larger class of energy sources with large levels of external stress resistance. Highly flexible devices facilitate conformal bonding with the non-planar substrates. Researchers employed several strategies for developing stretchable electronic devices that rely on translating stretching movements into microscopic-level bending tension. We can model the peak pressure associated with a thin layer during bending motion, according to the following equation [53]:

(1) $$\varepsilon =\left(\frac{d_{f}+d_{s}}{2r}\right)\times 100\%$$

where ‘*ε*’ denotes the peak strain, ‘*d_f_*’ and ‘*d_s_*’ represents the thickness of the film and the substrate, respectively, and ‘*r*’ is the radius of curvature. 

The flexibility of the devices can be augmented by transforming them into a stretchable type. The bending strains can be converted into tensile strains by incorporating buckling and coiling features to the flexible devices. Most of the conductive metal thin films are flexible to some extent with the radii of curvature of a few millimeters along with a flexible substrate.

Percolated thin film formation is one of the strategies used to merge conductivity and stretchability. The intentionally fractured thin films of graphene, carbon nanotubes (CNT), or thin metallic films can be structured on the elastomeric substrates [54]. During straining, the conductive layers fracture into plates which results in the formation of percolated networks. Another simple strategy is to embed the rigid active materials into elastomeric polymers to produce stretchable devices. The high volume fractions of nanosized conductive metal particles like silver [55], other carbon materials such as CNTs [56], or a composite of CNT-Ag [57,58] materials are generally impregnated with the stretchable polymers. These intrinsically elastomeric devices can withstand enormous strain and sustain their energy storage or generation performance without affecting their electrical conductivities.

The incorporation of buckled thin films is another approach for attaining highly stretchable electronic devices [53,59,60]. The films can be prepared on pre-strained elastomeric substrates by various thin film deposition techniques such as vapor deposition [61]; thermal evaporation [62], pulsed laser deposition [63], etc., and the releasing of pre-strain generates 3D buckled patterns which undergo bending during mechanical stretching agitations, thus, protecting the device from cracking [53]. The buckling can be established by bonding thin films under low and high applied strain; the films can be attached only at the active regions of the substrate whereas the rest of the regions are freestanding. Under a small amount of applied pressure, we can model the buckling wavelength according to a direct proportionality between Young’s modulus of the layer (*E_s_*) and the substrate (*E_f_*) and thickness of the film. For elastomers, when high strain values are applied, the buckling wavelength is dependent on the strain. In this case, the Young’s modulus, the thickness of the film, and the buckling wavelength (λ) are related through Equation (2) [53]:

(2) $$E_{f}=3E_{s}\left(\frac{1-V_{f}^{2}}{1-V_{s}^{2}}\right){\left(\frac{\lambda }{2\pi d_{f}}\right)}^{3}$$

where *V_f_* and *V_s_* are the values of the Poisson ratio of the film and the substrate, respectively.

Merging conductivity and transparency is another coveted goal of the researchers to realize transparent elastic conductors, which can find applications in invisible power sources or transparent wearable displays. Soft materials like conductive polymers [64], carbon nanotubes (CNTs) [65,66], graphene [67], metal nanoparticles [68] and metal nanowires [69,70] are extensively utilized to develop transparent flexible electronic devices.

## 3. Wearable/Flexible Architectures and Materials for Fuel Cell and Batteries

### 3.1. Polymer Electrolyte and Zinc–Air Fuel Cell

Notwithstanding the high conversion efficiency, zero emissions and high specific area power density (up to 1.6 W cm^−2^ or higher [71]), traditional proton exchange membrane fuel cells (PEMFC) [72] are not suitable to be adapted in flexible devices due to the rigidity, weighty and cost of the graphite plates and current collectors as well as to the equipment required to feed oxygen and hydrogen to the electrodes. The simplification of structure, materials, operations, and architectures of PEMFCs has been the object of intense research in the last decade. The strategies used by researchers to innovate the inflexible components of PEMFC were focused on the developments of soft materials and flexible configurations able to transform traditional into bendable devices for wearable applications, such us customer electronics [73], smart clothing [74] and healthcare [75,76]. Most of the literature working on the fabrication of flexible current collectors and electrodes is focused on the use of highly conductive and stretchable materials, such as metal nanostructures [68,69,70], conductive polymers [64], graphene sheets [67] and CNTs [66,67]. Conductive materials are often embedded insoft carbon-based materials [77] and/or polymer endplates [78,79] used to provide mechanicalsupport to the whole device.

One of the first approaches to this topic was proposed in 2009 by Tominaka et al. [79], which used a flexible cyclo-olefin polymer substrate, instead of brittle and expensive silicon, to fabricatethe on-chip fuel cell, a new class of bendable fuel cell. Their concept is based on a fuel cell with a scotch-tape structure that can be pasted everywhere and tailored for specific microdevices. Gold current collectors are deposited by electron beam evaporation on the polymer after adhesiveness improvement by green oxygen plasma treatment. The low surface area of the electrodeposited Pt-Ru catalyst layer and the related low delivered power density are major drawbacks of this novel device. Therefore, in the subsequent years, intense and appealing research has been devoted to the development of more successful electrodes, electrolytes, and support bendable materials and cell configurations capable of electrochemical performance adequate to wearable power sources.

In current flexible devices, the electrodes are required to possess high specific surface area, electrical conductivity, and excellent bending resistance. The materials usually employed in this scope are based on carbon nanotubes (CNTs) [80], graphene [81,82], conducting polymers [83,84], silver nanowires [85] and carbon cloth [86].

Silver nanowires (NWs), especially in their percolating structure, have been rigorously investigated to this scope because of their high mechanical stability and the ability to maintain a stable conducting path during bending and stretching under high mechanical stress [87,88]. In these studies, larger-area highly bendable fuel cells fabricated with Ag nanowire-based current collectors on flexible endplates made of polydimethylsiloxane are reported to provide high performances that surprisingly increase with the bending radius. These interesting results are attributed to the beneficial effects of the bending pressure on the contact and charge transfer overpotentials. Further improvement of the fuel cell output has been obtained by submitting the fuel cell to mixed bending and twisting mechanical load [89], and by engineering the stiffness of monopolar plates [90].

**Figure 1:**
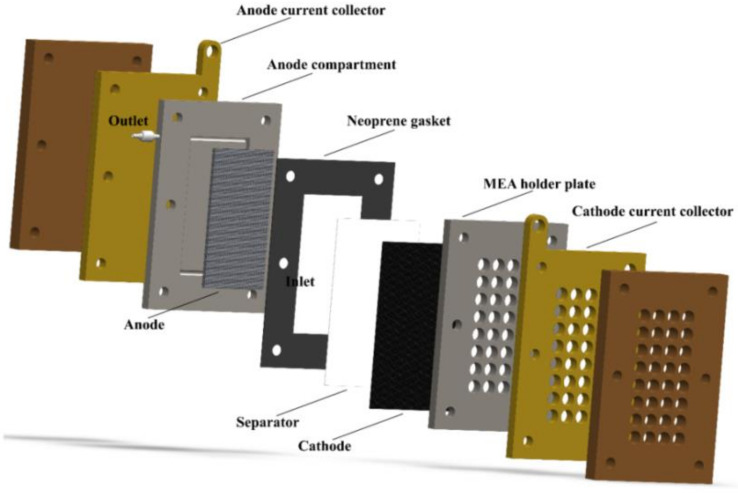
Exploded view of fuel cell components in an air-breathing configuration. Reproduced from reference [93] under CC BY 4.0 license.

Flexible composite electrodes composed of CNT membrane and carbon paper give also successful results in terms of electrical conductivity, flexibility, and gas permeability, as proven by the novel light and flexible PEMFC studied in reference [91]. The authors also designed a novel arrangement by totally replacing cathode graphite plates with a light (0.065 g), flexible, and cheap plastic membrane obtaining higher energy density than conventional PEMFCs. In the cathode design, they used the air-breathing concept (Figure 1), introduced in 2013 [92], and afterwards, intensely developed to make PEMFCs suitable to wearable devices [93].

**Figure 2:**
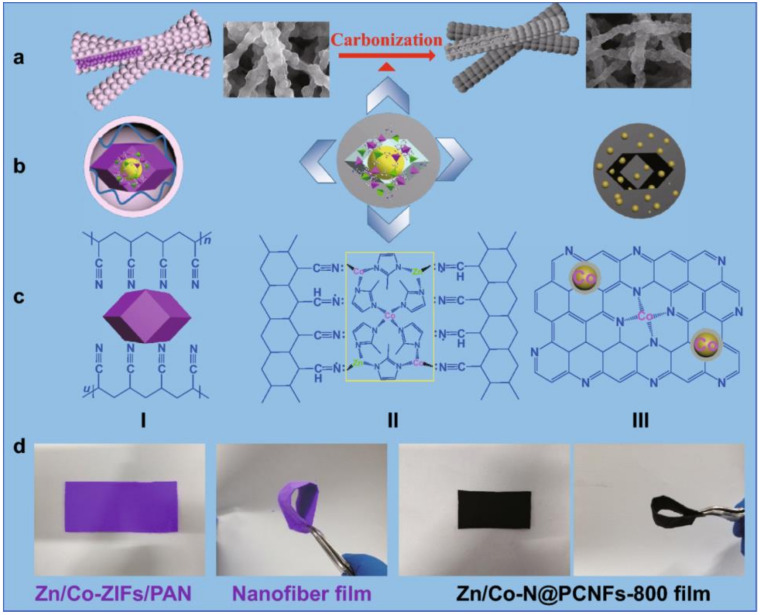
Schematic illustration of (**a**) sketch of the structures and SEM photos of the nanofibers, (**b**) simulated cross-sections, (**c**) simulated molecular structures, (**d**) digital photographs of the Zn/Co-based nanofibers film before and after direct carbonization at 800 °C. Reproduced from reference [95] under CC BY 4.0 license.

Another important issue of this topic covers the choice of suitable oxygen reduction reaction (ORR) electrocatalysts, which are known to be key materials for efficient and highly performant PEM and Zn–airFC devices [82,84,94]. To address this issue, novel synthetic strategies have been proposed for flexible cathode fabrication based on the electrocatalytic metal–organic framework (MOF). For example, metal-doped carbon nanofibers with superior ORR catalytic activity were fabricated by Niu et al. [95] through direct carbonization at 800 °C of electrospun Zn/Co-ZIFs/polymer nanofibers (Figure 2). The obtained ORR MOFs provide ORR superior performance in terms of onset (0.98V vs. RHE) and half-wave potentials (0.89V vs. RHE) as well as limiting current density (5.26 mA∙cm^−2^), than that of traditional Pt/C mixtures.

**Figure 3:**
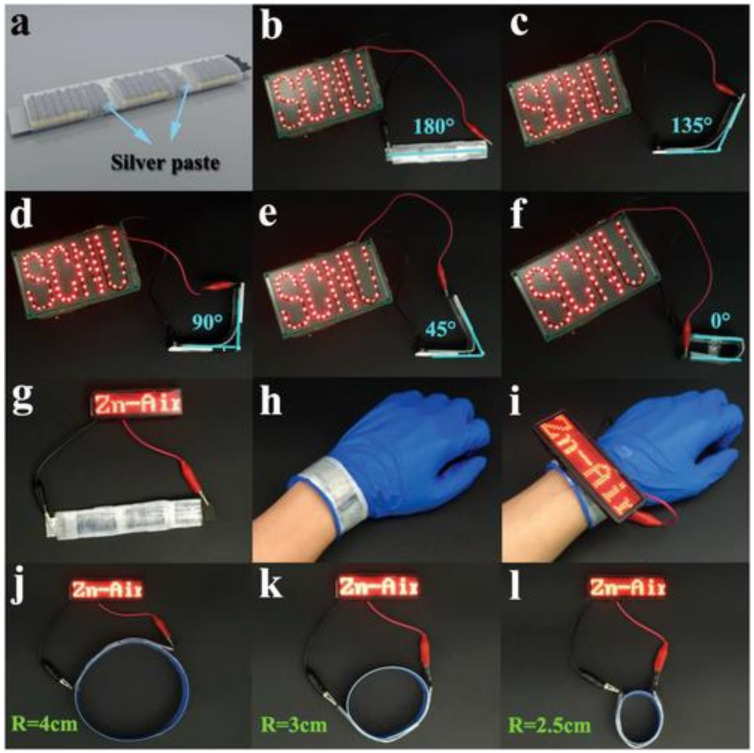
Digital photos of (**a**) a flexible solid-state zinc–air battery (ZAB) with three modules in series; (**b**–**f**) 75 red LEDs with “SCNU” shape powered by the ZAB of the panel (**a**) under bending angles of 180°, 135°, 90°, 45°, and 0°, respectively; (**g**–**l**) ZAB under bending angles of 0° powering a 3V LED breastpiece, ZAB as a wearable bracelet, ZAB powering a 3V LED breastpiece at different bending radii of 4, 3, and 2.5 cm, respectively. Reproduced from reference [96] under CC BY 4.0 license.

Another example arises from hierarchical 3D and 2D cobalt-based MOFs OER/ORR electrocatalysts deposited directly on a carbon cloth substrate through an easy carbonization-oxidation process [96]. The obtained electrocatalysts assembled in the flexible zinc–air battery (Figure 3) possess excellent flexibility as well as outstanding activity and stability.

The simplification of PEMFC structure and assembly needs also the selection of appropriate soft materials to be used as a substrate and a proper configuration that minimize electron and ionic pathways. A simple approach is the direct integration of current collectors to the solid electrolyte membrane through a support material. Many FC devices have been fabricated by using polymeric or photosensitive glass substrates with the main sufferance of inefficient designs with enormous electrochemical inactive areas. A solution to this problem is provided by Zhu et al. [97], who use a support material made of Silicon-On-Insulator (SOI) wafers filled with Nafion with current collectors obtained by gold sputtering. This design allows a more efficient device with good power densities thanks to the large reduction in electrical and ionic overpotentials. Another contribution provides a novel concept of flexible polyimide membrane with bi-conductivity [98]; electrical conductivity is guaranteed on both sides of the membrane, while ionic conduction through its thickness. In this frame, innovative and easy in situ methods of synthesis of polymer proton-conducting membranes should be investigated to improve the contact resistances between the ionic and the electronic part of the fuel cell. Very recently, in situ ionotropic gelation of chitosan has been proposed as an efficient method (named BIG) for the preparation of green, soft, and highly proton conductive polymer structures [99,100]. Further development of this procedure is expected to improve the efficiency of flexible and wearable fuel cells.

Finally, an important unsolved issue in the development of flexible hydrogen fuel cells is represented by the rigidity and weight of the hydrogen storage tank. Recently, a flexible hydrogen generator able to both store and produce hydrogen under mechanical bending or rotating has been successfully proposed [101].

A strong simplification of the fuel cell materials has been also obtained by combining air-breathing and microfluidic concepts to obtain flexible microfluidic fuel cells, where the polymeric proton-conducting membrane is removed and substituted by liquid fuels and oxidants. As discussed in detail in Section 4, this configuration is highly exploited in the biomedical field, because sweat, urine, or human blood can be used as fuel due to its content of lactic acid, urea, and glucose, respectively.

### 3.2. Batteries

The development of wearable and implantable electronics with continuous and stable functioning strictly depends on the progress of power sources toward miniaturization, stretchability, bendability, and adaptability to the final wearable device. Material science aspects are crucial in the development of highly efficient wearable power sources. In this section, we report and discuss the research progress on wearable Li-ion, Li–S, Zn-ion, Zn–air batteries in terms of materials and related lightweight, flexibility, and electrochemical performances [102,103,104,105,106].

#### 3.2.1. Lithium-Ion Batteries (LIBs)

Lithium-ion batteries are currently preferred in wearable devices because of their environmental-friendly character and superiority to other power sources in terms of electrochemical performance (3.7 V), theoretical capacity (3860 mAh·g^−1^) together with a long cycle life and great safety. Materials science aspects of electrodes, electrolytes, and supports and their engineering to fabricate flexible batteries can be found in several reports [107,108,109,110,111]. The electrodes are the central components of the electrochemical battery because the kinetics and thermodynamics of the redox half-reactions occurring at their surfaces often govern the kinetics of the global process, i.e., the deliverable power. Typically, LIB electrodes are fabricated by coating a mixture of electroactive materials, polymeric binders, and carbon black to provide electrocatalysis and electric conductivity at the same time on the current collector (Cu and Al are the most used). Carbon-based soft materials with versatile dimensionality such as carbon nanotubes, graphene, and polymers possess excellent properties, including high electrical conductivity and excellent mechanical performance, which are recognized as the most suitable materials to enhance conductivity and support electroactive flexible electrode materials [112,113,114]. They succeed to convert typical non-conductive flexible materials suitable for wearable energy storage design (such as conventional paper) into highly conductive components. In LIBs, layered carbon-based material and lithium alloys are generally employed as anode materials, where the insertion and extraction of Li-ions occur at ∼0 V vs. Li/Li^+^ electrode; however, for the most part, research studies have been focused on cathode materials, where the intercalation and de-intercalation of Li^+^ occur at a potential of ∼4 V vs. Li/Li^+^ electrode (for example LiCoO_2_, LiMn_2_O_4_, and LiFePO_4_).

To obtain great flexibility, the fiber-type electrode concept is often utilized. Fiber electrodes with outstanding flexibility, conductivity, and mechanical resistance can be fabricated by assembling CNTs and 1D–2D graphene for flexible Li-ion batteries [115,116,117]. Many interesting examples of CNTs structures tailored to textile electrodes can be found in the literature [118,119,120,121]. One of them is provided by a coaxial fiber-shaped lithium-ion battery [117,120]. Weng and co-workers [120] deposited CNT/Si and CNT/lithium manganate (LMO) composite yarn on a cotton fiber substrate to obtain a hybrid layered structure for the cathode and the anode, respectively. The yarn-based textile full LIB cell enables an areal energy density of 4.5 mWh∙cm^−2^ showing promising potential for portable and wearable electronics. To obtain lightened, flexible, and powered lithium-ion batteries for wearables, Ren et al. [122] removed from the battery current collectors and binders, designing a wire-shaped device with two aligned MWCNTs/lithium oxide composite yarns as the anode and cathode. Highly stretchable and conductive electrodes (up to 400%) can be also obtained by combining CNT sheets with polymers (for example PDMS) in a sandwich structure [123].

In addition to CNTs, graphene has been also widely used in wearable LIBs due to its superior electrical conductivity and mechanical strength. Xie and coworkers [124] showed that the presence of graphene increases the ability of carbon fiber cloth to support Co_3_O_4_ nanoparticles, enhancing the reversible specific capacity (400 mAh∙g^−1^ at 100 mA∙g^−1^) and the cycling stability of the resulting LIB anode. Metal nitrides are typically used to improve the capacity of flexible carbon electrodes for LIBs. In reference [125], flexible LIBs with superior cell performance have been fabricated by using 3D nickel nitride nanosheets deposited on carbon cloth by hydrothermal method. The results have shown that the nanostructured metal nitride-carbon nanosheets’ assembly can provide ultrafast electrode kinetics without ionic and ohmic overpotentials between the nanosheets. Liu et al. [126] obtained flexible anodes with similar performances by deposition of hierarchical nanostructured 3D ZnCo_2_O_4_ arrays on carbon cloth. The anodes are assembled with a LiCoO_2_/Al foil cathode and a LiPF_6_-based electrolyte. The design and materials used result in a highly flexible and very thin LIB device with greater electrochemical performance—a promise for large scale LED and displays wearable applications. Huang and co-workers [127] prepared N-doped carbon-based cloth by using 3D porous graphene and cotton cloth. They obtained superior flexible LIB anode with higher specific surface area, mechanical resistance, and electron conductivity. N-doped carbon nanofibers/carbon nanotubes [128] and Li_4_Ti_5_O_12_/polydimethylsiloxane sponge [129] flexible anodes showed excellent performance. 

Moreover, electrolytes and separators are considered to be important components in a stretchable system. Solid-state polymer and gel electrolytes are typically used because of higher safety and stability than conventional liquid electrolytes [130,131,132,133].

Crosslinked gelpoly(vinylidene fluoride-co-hexafluoropropylene)-based polymer electrolytes, with high tensile strength (10.6 MPa), excellent elasticity, and superior electrochemical stability, have been synthesized by Guan et al. [134]. Similarly, ionic liquids and Li-ions have been loaded into co-polymeric membranes to obtain ionogel structures with the ability to repair mechanical damage, good ionic conductivity, and flexibility [135]. These electrolytes tested in a Li/LiFePO_4_ battery produce a high discharge capacity of 147.5 mAh∙g^−1^ and excellent cycling performance. Crystal-polyethylene oxide-based [136] and succinonitrile-based gel polymer electrolyte [137] have been also successfully tested in lithium-ion batteries.

**Figure 4:**
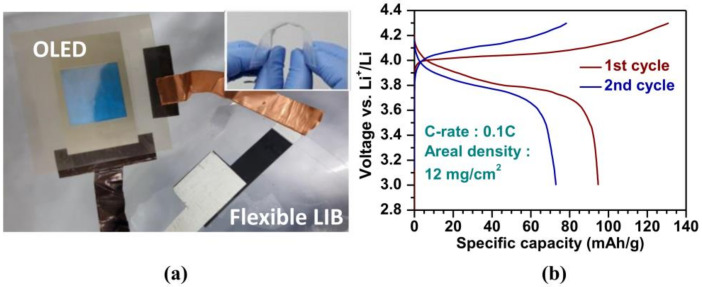
(**a**) Digital photo of a Flexible LIB cell that illuminates a blue Organic Light Emitting Diode (OLED) at 4 V; Inset: flexibility of the LIB cell under bending; (**b**) galvanostatic charge and discharge curvefor the flexible LIB cell. Reproduced from reference [138] under CC BY 4.0 license.

Another example is shown in Figure 4, where a flexible LIB based on gel polymer membrane made of poly(vinylidene fluoride-co-hexafluoropropylene) is reported [138]. The electrolyte provides better ionic conductivity (2.3 × 10^−3^ S∙cm^−1^) than conventional polypropylene separators due to the optimization of the crosslinked morphology.

The attention has been also focused on the optimization of ionic contact at the electrode/electrolyte interface. Shin et al. [139] fabricated a stretchable polyurethane/poly(vinylidene fluoride) membrane with an optimal ionic transport at the electrodes during stretching and bending. Good results under severe stretching deformation (270% strain) are reported for poly(styrene-b-butadiene-b-styrene) block copolymer based as a rechargeable LIBs separator [139].

The use of novel polymer, metal, and paper foils resistant to both bending and stretching has also been proposed for wearable rechargeable LIBs [140,141]. Finally, it is noteworthy the promising role of the LIBs origami battery concept in wearable devices due to the enormous linear and areal deformability, twistability, and bendability [142].

#### 3.2.2. Zn-Ion Batteries

**Figure 5:**
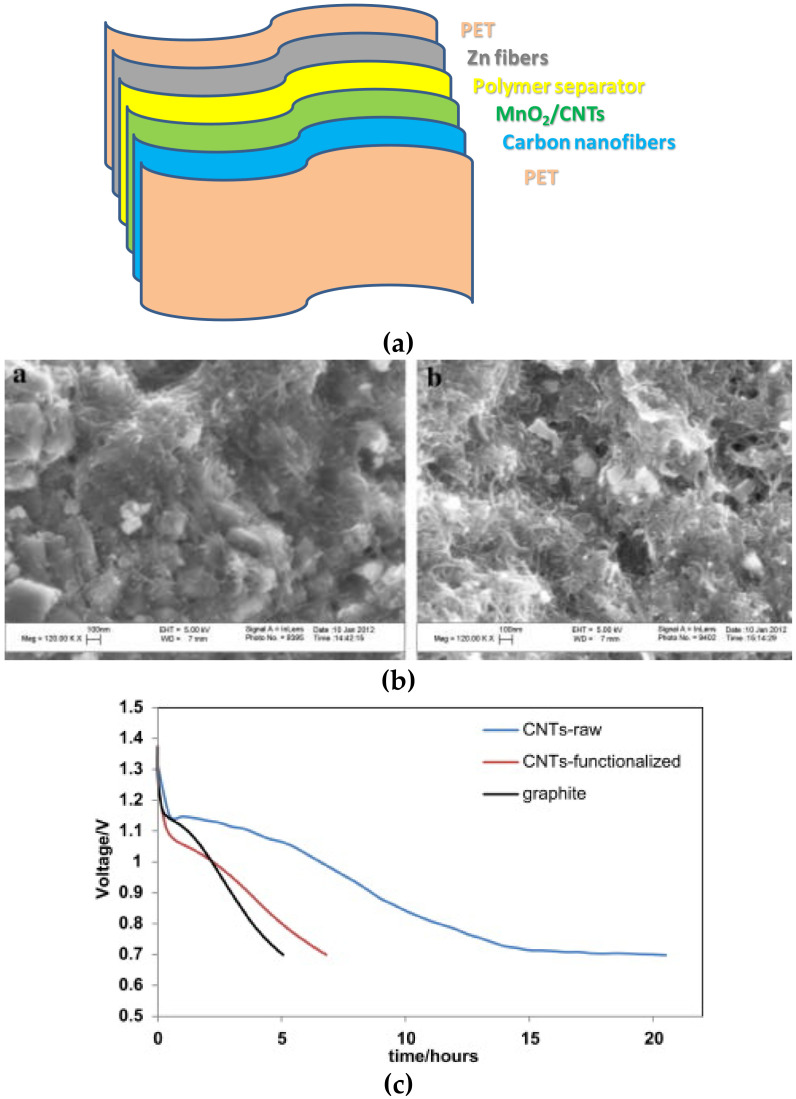
(**a**) Sketch of the typical components of a flexible Zn–carbon battery; (**b**) SEM micrographs of MnO_2_-cathode containing functionalized MWCNTs (left) and Zn particulate anode (right); (**c**) Zn–C discharge curves for different cathodes at the same load (8.6% *w*/*w*). Panels (**b**,**c**) are reprinted from reference [146], copyright (2013), with permission from Elsevier.

Flexible Zn-ion batteries are promising devices for wearable electronics thanks to their intrinsic advantages—safety, non-flammable, non-toxic, and low-cost [143,144]. As shown in Figure 5, flexible Zn–carbon batteries are typically constituted by a current collector made of carbon nanofiber mats, a cathode made of MnO_2_/SWCNTs, a polymer electrolyte constituted of polyethylene oxide containing TiO_2_ nanoparticles and an anode made of Zn foil [145,146]. This Zn–carbon battery maintains good power output under mechanical stress, showing good flexibility.

Many combinations of materials have been analyzed in the literature. By using a Zn/carbon fiber anode and Co_3_O_4_/Ni foam cathode with aqueous electrolyte, a flexible rechargeable battery with a high energy density of 241 Wh∙kg^−1^ and long-term cyclability has been fabricated by Wang et al. [147]. Various soft materials, carbon-fiber paper, and other flexible carbon-based materials with high conductivity are used as an alternative to the conventional metal current collectors, as detailed in Section 3.1. The use of flexible stainless steel mesh as the current collector is promising for wearable applications as solid-state Zn–air battery can deliver energy density of 847.6 Wh∙kg^−1^ and a cycling resistance of 600 h at 25 mA∙cm^−2^ [148]. By coating lightweight and low cost commercial carbon fiber current collectors with active electrode materials, Yu and co-workers [149] succeeded to fabricate a high discharge capacity (158 mAh∙g^−1^) zinc-carbon battery. The optimal flexibility and bendability of the device allowed the fabrication of batteries in series to feed commercial green light-emitting diodes.

Guan and co-workers [150] investigated the mechanical and electrochemical resistance of flexible Zn–air batteries based on 3D carbon nanotubes/a Ni foam cathode to severe bending mechanical stresses, obtaining good results. Similar studies on air-cathodes for Zn–air batteries have been conducted by Mu et al. [151] by coating C-fiber papers with P-doped graphitic carbon nitrides, obtaining highly ORR electrocatalytic flexible electrodes.

Flexible Zn–MnO_2_ batteries have been also developed by engineering MnO_2_ electrodes with poly-3,4-ethylenedioxythiophene as a buffer layer and PVA/ZnCl_2_/MnSO_4_ gel as the electrolyte. The obtained great capacity (366 mAh∙g^−1^) and energy density (504.9 Wh∙kg^−1^) together with good cycling performance make them promising for powering portable and wearable electronics [152].

Despite several studies that have provided an optimal choice of materials combination and architectures, the fabrication of environmentally friendly and safe wearable fiber-shaped aqueous rechargeable batteries (FARBs) is still challenging. By removing toxic and unsafe components, Guo and co-workers developed an eco-friendly and highly safe wearable rechargeable Zn battery based on a soft pyrene-based cathode [153]. Besides, a prototype of safe, low cost, and wearable energy storage coaxial-fiber Zn-ion battery (named CARZIB) with performance (195.4 mWh∙cm^−3^ at a power density of 0.2 W∙cm^−3^) comparable to a conventional Zn-ion battery was fabricated by Zhang and co-workers [154]. The coaxial structure is composed of a core axial anode made of Zn nanosheet on carbon nanotube fibers and a cathode of ZnHCF composite on aligned CNT sheets at the external surface. The two electrodes assembled on a ZnSO_4_ gel electrolyte in a coaxial shape provide small charge transport and contact ohmic overpotentials as well as advanced mechanical flexibility and performance stability under long-term bending.

It is known that the choice of organic electrolytes provides a solution to the safety issues of LIBs and conventional aqueous electrolyte-based batteries. This aspect becomes particularly highlighted in wearable devices because the organic composites may also provide high flexibility. For example, electrolytes based on zinc triflate combined with polymers can improve the low conductivity and degradation issues of non-rechargeable primary batteries [155]. The safe crosslinked porous gelatin-based electrolyte has been discovered by Li and co-workers [156] and tested in a ZIB with excellent power and energy performances. In addition, this battery possesses high resistance under several environmental conditions typical of wearable devices (for example, being waterproof), making its application promising for a commercial smartwatch or wearable sensors. Very recently, Pan and co-workers [157] developed a fiber-based ZIB with a CNT-zinc pyrovanadate cathode to greatly improve electronic conductivity and mechanical robustness of the full device.

#### 3.2.3. Li–S Batteries

Lithium–sulfur (Li–S) batteries have received much attention due to their multielectron chemistry, multiphase, and multistage nature of the electrode reactions, superior capacity, and a high theoretical energy density of 2597 Wh kg [158]. In wearable Li–S batteries, elemental sulfur and lithium undergo reversible electrochemical reactions in an organic electrolyte as in conventional devices. The difference resides in the necessity of relevant procedures able to convert sulfur and lithium metal in flexible electrodes and solid-state polymer electrolytes.

**Figure 6:**
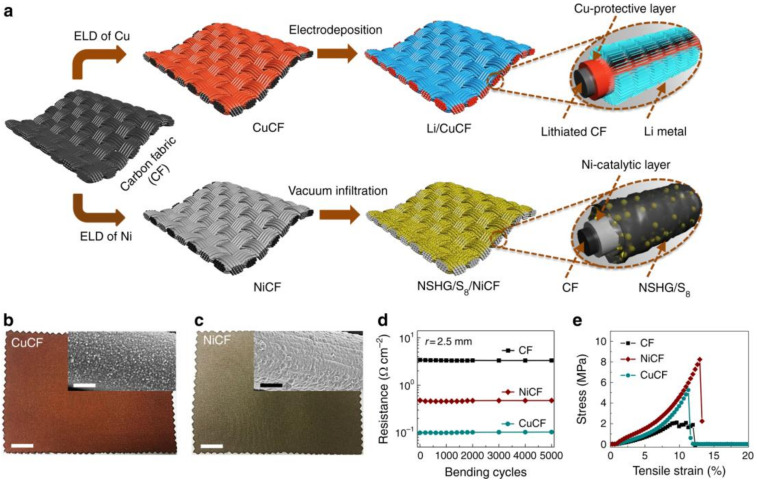
(**a**) Fabrication process of lithium and sulfur electrodes from carbon fabrics (CF); (**b**,**c**) digital (scale bar = 3 cm) and SEM (scale bar = 3 µm) photographs of CuCF and NiCF electrodes; (**d**) CF, NiCF, CuCF resistances and (**e**) tensile stress–strain curves. Reproduced from reference [172] under CC BY 4.0 license.

Flexible sulfur cathodes are typically constituted on carbon materials as well as the other power source electrodes (see also the Introduction Section). Notably, various excellent research works deal with S-based cathodes for wearable applications by depositing or anchoring sulfur to flexible carbon cloth/paper [159,160], CNTs [161], graphene [162], carbonized polymer [163] and polymer-glue [164]. For example, 3D-hierarchical composite cathodes for flexible Li–S batteries synthesized via sulfur encapsulation show high tensile strength twice that of the original carbon cloth retaining bending resistance to 200,000 cycles [165]. The obtained Li–S battery is extremely versatile to different sizes and shapes of fabrication, demonstrating high potential for wearable applications. Among the different studied geometries, dual core-shells based on N-doped graphite and a cable-shaped sulfur cathode show promising results [166]. Another approach is based on the choice of highly porous carbon electric conductive supports able to load great quantities of sulfur. For example, Chong et al. [167] fabricated a sulfur flexible electrode by filling a composite reduced graphene oxide/CNTs fiber electrode with a large amount of sulfur with liquid crystalline behavior [168]. With the same aim, highly porous and conductive graphene foam [169] and porous graphene/sulfur composite ribbon electrodes are used to fabricate cable-shaped lithium–sulfur batteries [170]. Commercial wearable stainless steel fibers have been also successfully tested as sulfur supports and current collectors [171]. In Figure 6, the use of metal-coated carbon fabrics (Cu-coated for the anodic and Ni-coated for the cathodic side) as support of Li and S is reported [172]. The procedure allows fabricating Li–S full cells with high energy density, areal capacity (3 mAh cm^−2^), and cycling stability for more than 200 cycles with good resistance at small curvatures.

Many other research efforts have been made to develop wearable/flexibleLi–S batteries; for example, other interesting improvements can be found on cathodes [173], current collectors [174] and electrolytes [175].

## 4. Wearable/Flexible Architectures and Materials for Biosensors and Biofuel Cells

In terms of implantable/wearable electronics, the most promising applications will be in the human healthcare field related to personalized medicine, point-of-care diagnosis, health conditions monitoring/screening, self-powered prosthetics, and health devices [176]. Differently from the other possible devices [177], biosensors and biofuel cells can offer a more compact, self-contained, and biocompatible option [178]. These devices are based on the strong substrate specificity and selectivity of enzymes and the ability of some microorganisms to convert organic molecules. These processes generate electrons that can be harvested by an anode and conveyed to an external circuit to power microdevices [179] or to collect electrical signals directly related to the concentrations of analytes [180,181] or biomarkers [182]. Applied to human bodies, especially for the case of enzymatic fuel cells (EFCs), some of the molecules (e.g., glucose, lactate, ascorbate, etc.,) present in physiological liquids (e.g., blood, tears, sweat, saliva, etc.,) are the energy source for these devices [183]. In the beginning, implantable EFCs gained attention and interest [178], but the invasive nature and the biocompatibility issues of these devices retarded their progress, which should have kept the pace of faster development in wearable/flexible devices. The case of microbial fuel cells (MFCs) is slightly different and more devoted to biosensing, detection of toxicants [184], and treatment of pollution agents in water sources [185], with energy harvesting from liquid wastewater [186] or solid waste biomass [187], which contain many biodegradable molecules. In addition, in MFCs, there is nowadays a significant development of the concepts of flexibility, one-use, and disposable devices, related to the materials employed, which will be covered below.

For the basic description and advanced understanding of the chemistry, performances, and applications of these devices, not intended to be covered in this review, the readers are invited to check the most recent contributions to the field, for example [187,188,189,190,191,192]. In the following sections, emphasis and criticism will be put on the architectures, supports, and soft materials used to obtain wearable and flexible devices by using advanced techniques and to show case benchmark studies in EFCs and MFCs. The aim is to critically introduce and explain the essential concepts and practical techniques involved in the topic and to give simple, clear, and useful information to those who want to approach this field in an innovative way, rather than just exposing, citing or listing the existing literature.

### 4.1. Architectural Concepts, Structures, and Supports for Enzymatic Fuel Cells

Enzymatic Fuel Cells (EFCs) are usually membraneless devices, if not indicated otherwise, and the abiotic components are represented by the electrode materials for anode and cathode, any eventual redox mediator, the external case or the support representing the hard/soft structure of the EFC, and the current collectors/wiring [193]. The biotic components are the enzymes employed at the anode and/or cathode and any eventual cofactor needed to carry out the enzymatic reactions at the electrodes [180]. These components determine the biochemistry and the generation of the electrons in the device, whereas the abiotic components rule over the harvesting and transport of the electrons from/to the enzymes through the electrodes, provide the connection with the external circuit, and give the basic mechanical flexibility/robustness to the whole device.

It is important to keep in mind the list of the essential biotic/abiotic components because the final architecture of the wearable EFC will be the synergic assembly of these components. Even if from the conceptual point of view, the selection of the enzymes and cofactors related to the target substrate in the physiological fluid to be exploited is the first step, this is a tied choice because the most convenient combinations of enzymes, cofactors, and substrates are already known, and the choice is limited [194]. It should be also stated that these delicate biotic components, due to their easy degradation, leaking, long-term stability, and denaturation issues [195,196], must be applied to the EFC as the last ones by steps and procedures not involving warm temperatures, extreme pHs, and aggressive washings. Impregnation, layer-by-layer, air-drying, and weak interaction-based orientation methods are used for the fixation of enzymes [197,198].

Therefore, in the vision of wearable/flexible EFC realization, a more important step is the selection of a relevant support where the rest of the device will be built, because if the support is flexible, stretchable, and lightweight, the device will adapt well to the human body or skin when operated. This concept is simple but not trivial and it is indirectly suggested in the literature review.

**Figure 7:**
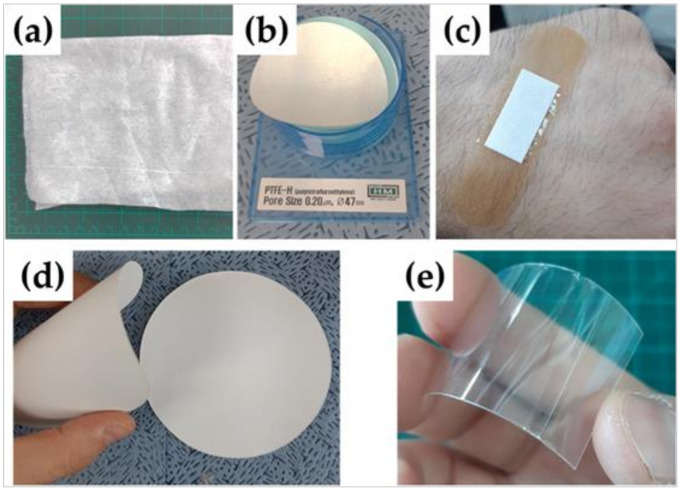
Examples of flexible supports for wearable EFCs that can be easily found in the lab: (**a**) textile fabric; (**b**) a polymeric filter disk; (**c**) a medical skin patch; (**d**) a filter paper disk; (**e**) a flexible transpirant plastic sheet. Many other flexible/stretchable supports were used in the literature.

In many works, it is possible to observe that the wearable EFCs were built starting from paper [199], textile fibers [200], patches [201], other bendable plastic objects [202,203], or directly on the skin as a tattoo [42,204]. Therefore, the very first requirement for wearable/flexible EFCs is a soft and dielectric structural support (Figure 7).

**Figure 8:**
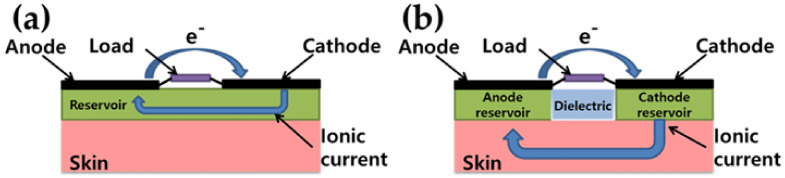
Possible architectures for skin EFCs with reservoir: (**a**) one reservoir, conductive; (**b**) two separated reservoirs with dielectric separation for the ionic current through the skin—a porous transpirant microlayer at skin/reservoir interphase may be included.

Due to the specificity and selectivity of the enzymes, another peculiar feature of EFCs is the almost complete absence of crossover issues, and therefore, separation membranes are not needed. A structural consequence of this detail is that the electrodes can be realized in close proximity to each other (but without any electrical connection to avoid electrical short circuits) and on the same side of the support. If the device is intended to be applied on the skin, for example, this means that both electrodes will face the skin; the availability of the substrate and of the terminal electron acceptor (TEA) to supply the electrodes must be carefully taken into consideration. To partially avoid this problem, there are some architectures proposing devices with a built-in reservoir, to work as a finite biobattery [204,205] or as a timer dispenser [201] (see Figure 8).

The built-in reservoir is usually another relatively thick layer fully impregnated with the substrate, preferably a gel or a hydrogel, representing a very interesting challenge to be faced.

To maximize the specific surface area for enzyme loading and the contact area of the electrodes, the common structure adopted to realize the wearable EFC includes multiple thin layers with specific functions, like a basic conductive layer for current collection (e.g., gold, platinum, conducting polymer, etc.), a nanoengineered highly porous layer with a large surface area (e.g., carbon nanotubes, graphene nanosheets, carbon or metal nanoparticles, etc.,) for enzyme and cofactor immobilization, another layer for mediator/crosslinker reservoir, and eventually, a final ion-conducting layer (e.g., Nafion 117 solution) as protection from leakage and any damage from the external environment [206,207]. The combination of these layers makes the final architecture of the EFC; if connected in space by series or parallel, they can determine various compact designs and stacks. This is the reason why dense electrodeposited, sputtered or roll-to-roll patterned metal layers are the best ones to be used as current collectors/wire terminals on the flexible support as the base for the other layers [208,209].

The stretchable/bendable/flexible properties of the other layers are determined by the “softness”, connectivity, and mechanical adhesion of the materials employed. These layers are obtained with optimized deposition, printing, or painting processes, and their optimization and sequence is a very interesting and still open field for research, as well as the formulation and preparation of the soft materials in the form of liquid precursors to be applied on the support.

### 4.2. Soft Materials for Enzymatic Fuel Cell: Inks, Crosslinkers, and Embedded Mediators

The abiotic materials used in wearable EFCs are regarded as “soft” in the sense that they are not bulk solids with remarkable sizes and dimensions, but thin layers, very often including nanosized components blended with specific chemicals, obtained from liquid solutions, and therefore, possess somehow flexibility, adhesion on curved and irregular surfaces, resistance to shear and stress [210]. The thickness of these layers is a fundamental parameter, and it is a function of the particular deposition method used other than, obviously, the amount of precursor and the shrinkage upon drying (i.e., vol% of the volatile solvent employed).

**Figure 9:**
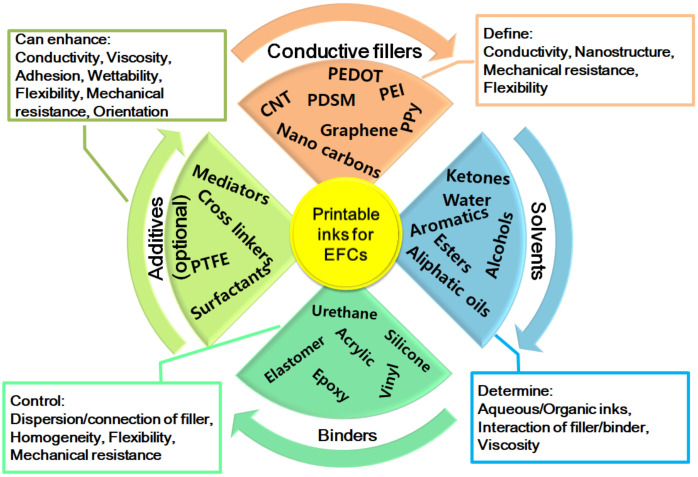
The four components for the advanced formulation of printable inks as the main soft material for wearable EFCs. The role and specific properties affected by each component are reported.

The realization of these thin functional layers, mainly the electrodes, is based on ink technology. As the fabrication of wearable EFCs is largely based on printing processes, the formulation of the printable inks used on the flexible supports determines the final mechanical performance of the layers and the electrochemical performance of the device. Printable inks for EFC electrodes have four major components: the conductive filler(s), the binder, the solvent, and the additive(s) (see Figure 9).

The solvent is the liquid basis part and imparts the separation between aqueous and organic inks. Other than simply water, many organic solvents are used in ink formulations (e.g., alcohols, esters, ketones, aromatics, etc.), and the proper choice is led by the desired/possible interactions with fillers and binders, which determine the flowability, viscosity, and homogeneous dispersion of the components over the printing support. The cohesive energy between solvent, filler, and binder can be estimated in advance by using the Hildebrand–Hansen parameters [211,212], thus, modeling and preparing homogeneous, flexible, and printable inks.

This is important to enable the binder to do its job—a polymer material is employed to keep dispersed but connected, in a homogeneous way, the conductive filler upon solvent evaporation. This component is polymeric only and can be based upon acrylic, urethane, elastomer, silicone, vinylic and epoxydic polymers, depending on the type of functional groups on the conductive filler to have a specific reaction or interaction with it. The efficiency and stability of the binder, together with proper dosage, affect the macroscopic homogeneity of the layer and the microscopic connectivity of the filler to ensure high conductivity, high density, and high mechanical resistance.

The main active component of the ink, for which the others are selected for, is the functional or the conductive filler. While the solvent and the binders are almost exclusively organics, the filler can be metallic, ceramic, or plastic/polymeric, depending on the desired application. In the advanced inks used for wearable/flexible EFCs, the conductive filler is also nanostructured and functionalized, tailor-made to meet complex and multiple requirements. This is the most variable component to be studied because a large variety of fillers has been used so far. Especially for EFCs, the conductive nanofillers for inks are represented by carbon nanomaterials, e.g., graphene, carbon nanotubes, carbon nanosheets, carbon nanowires/fibers, etc., [213,214,215] or conductive functionalized polymers, e.g., poly(3,4-ethylenedioxythiophene): polystyrene sulfonate (PEDOT: PSS), polyethyleneimine (PEI), polypyrrole (PPy), and polyindole [198,216,217,218]. The first ones have very high conductivity, mechanical resistance, and highcost, while the latter have low cost, softness, flexibility, easy manipulation, and good conductivity. Carbon nanomaterials and conductive polymers could be used together in a mixed way or alone to tailor costs, conductivity, and flexibility.

The last component is a generic one called an additive; it is like a jolly because it can be selected to impart an additional feature to the ink or to modify a basic property like viscosity, conductivity, flexibility, mechanical resistance, etc. It is generally an organic or polymeric molecule with a specific function, different from the filler. For example, it can be a surfactant to give additional dispersion/orientation of the nanosized filler, a hydroxylated molecule to increase wetting and polarity of the ink, or a polymer with high molecular weight to increase viscosity. In this regard, for EFCs, the most important aspects are enzyme immobilization [219,220] and electron transfer, so the insertion of an additive capable of providing inks for layered electrodes with better enzyme loading/retaining and mediated electron transfer is greatly desired. From the literature, an improved enzyme immobilization can be obtained by using crosslinkers, e.g., glutaraldehyde, terephthaldehyde, naphthalene thiol [221,222], which could be employed as additional components in inks, whereas the electron transfer can be updated by embedding a redox mediator, e.g., neutral red, methylene blue, Prussian blue, tetrathiafulvalene [204,205,223,224], directly in the electrode layer through the ink. There are recent examples of how a mediator can be bonded to carbon nanomaterials, i.e., a conductive filler, by a layer-by-layer procedure [224] or blended with carbon nanotubes in inks [42,204]. The use of an additive is not mandatory for ink formulation, but it could be useful to modify existing recipes to obtain printed layers with unusual properties or unique functions, starting from commercial formulations of inks for printable electronics, already available [225,226,227].

### 4.3. Flexible, Miniaturized, and Disposable Microbial Fuel Cells

Differently from EFCs, in MFCs, the biotic component is represented not by a single active and complex protein (i.e., an enzyme) but by a whole-cell, with its cellular membrane, cytoplasm, and organelles representing a living microorganism, usually organized into a social community known as biofilm [228]. The peculiar selectivity and specificity of EFCs are, thus, lost because the living cells of the MFC’s biofilm have a plethora of different enzymes to perform metabolic and catabolic complex bioreaction cycles, and not just a single and direct reaction. Usually, the microorganisms and bacteria employed in MFCs are not biocompatible with epidermal and human body applications with a wearable purpose [210], and this is why their application is more in the fields of wastewater treatment and environmental sensing rather than healthcare.

However, given a wide variety of residual biomass substrates, MFCs can harvest energy and produce electricity. As a potential power source for small scale applications, the miniaturization and portability of MFC devices is a frontier field of the last ten years [229], deserving increasing efforts still nowadays. Chambers’ volume miniaturization, together with very thin and porous microelectrodes is the state of the art to obtain portable micro MFCs with a finite amount of energy, like alkaline batteries, but high power density by volume. Fraiwan et al. [230] early demonstrated that for micro MFCs, especially the anode material for the biofilm’s housing, the materials should be 3D nanostructured (e.g., carbon nanotubes, nanofibers, etc.), chemically modified (e.g., gold nanoparticles/polymer complexes) to improve long-term durability. Structural complexity and low costlong-term durability are possible only by adopting advanced fabrication processes, like soft lithography for the case and skeleton of the device [231], and printable polymers, inks, and conductive polymers compatible with additive manufacturing techniques for the other components [232]. A very interesting and fruitful field should be the translation of ink technology used for EFCs to prepare MFC devices for testing, because from the fabrication and materials point of view, there are many commonalities with EFCs, apart from the necessity to consider also a separation membrane in the architecture of the device.

When long-term operations and the presence of a membrane are not strictly required, flexible and disposable MFCs are possible and successfully employed as biobatteries to power sensors or as biosensors themselves for discontinuous and one-shot use. These flexible MFCs are based on paper supports [233], usually laboratory filter papers, like Whatman and others, that can be patterned, printed, folded, and impregnated with the liquid electrode precursors, the inoculated anolyte and/or the catholyte including a mediator. In a recent work, Nguyen and Taguchi [234] demonstrated how it is possible to fabricate a ready-to-use paper-based MFC anode with a built-in dry biofilm by employing soft activated carbon sheets and a carbon nanotubes/mediator complex as a cathode with a sprayed selective separation layer as a membrane. Xu et al. [235] used a conceptually similar paper-based MFC as a biosensor for wastewater and highlighted that the use of paper increases the adhesion and growth of the biofilm due to the natural biocompatibility of cellulose compared to that of carbon cloth. Therefore, even if a paper is not by itself the conductive support, it can be flexible and biocompatible to enhance biofilm formation for micro MFCs. Recently, in another very important study [236], the role and managing of paper supports to prepare membraneless, 2D, miniaturized, and low cost reusable MFC sensors are explained. The procedure and recipe, including crosslinking of the paper foil, to realize the paper MFC is a good benchmark in the field and can be used as a basis for further improvements. Another architectural and unique feature of paper MFCs is that by mastering origami folding technique [237,238], it is possible to easily fabricate very compact microfluidic MFC stacks with cheap and soft materials. By checking and studying the recipes, procedures, and raw materials used in the mentioned works, it is possible to create new designs, architectures, and formulations for micro MFCs based on paper or other supports.

## 5. Wearable/Flexible Architectures and Materials for Supercapacitors

Supercapacitors are energy storing devices able to provide higher power density than fuel cells/batteries and higher energy density than conventional capacitors. These two features, together with their environmentally friendly nature, make supercapacitors significant in future energy storage applications; nevertheless, they need recharging to provide continuous long-term energy supplies. In recent years, the tremendous advances in bendable smartphones, flexible wireless, and biomedical sensors have attracted the scientists towards flexible wearable energy storing devices that give portability to the electronic devices.

Based on the charge storage mechanism, the supercapacitors areclassified into an electrochemical double-layer capacitor (EDLC) or pseudocapacitance supercapacitors. In EDLC, different kinds of carbon electrodes with extremely high surface area and conductivity are used because they require the storage of charge electrostatically. In contrast, pseudocapacitors need materials able to store charge faradaically, such as transitional metal oxides and conducting polymers that undergo some redox reactions during functioning. Significant research has been done on conventional supercapacitor electrodes and electrolyte materials, such as conducting polymers, metal oxides, carbon nanotubes, and activated carbon [239,240,241,242,243,244].

Therefore, due to high power density, long cycle life, rapid charging–discharging rate, and good safety wearable supercapacitors are promising candidates to fulfill future ambitions of flexible and wearable energy storage devices. Depending upon the method opted for, different geometries of wearable supercapacitors can be easily obtained like planar, fiber, and wire-shaped. One of the best ways is to develop fiber and make a cloth that can store energy. We can say that the electronics and textiles industries have to go together to achieve good performing wearable supercapacitors [245,246,247,248,249]. The textile electronics industry is growing very fast in the world and it is predicted that it will reach up to US$ 9.3 billion by 2024 [250]. The textile industry production includes the insertion of electronic gadgets, sensors, and displays in consumer clothing. One of the ways to boost the textile industry is to develop energy storage devices in the constituents of cloths, such as fibers. Fibers must contain all the components of traditional supercapacitors, such as electrodes, electrolytes, separators, and current collectors.

The energy storing fiber should have the three-dimensional properties of electrochemical function, non-electrochemical function, aesthetic, and lifecycle. Thus, fiber should possess high power and energy density and good capacitance. It should also be comfortable, flexible, and attractive in color. What should be also considered is the environmentally friendly property of the resultant wearable supercapacitor. Significant research has already been done on high energy, power density, and capacitance wearable supercapacitors.

It is known that the performance of a wearable supercapacitor depends upon electrode and electrolyte materials and device configuration. However, the key role can be ascribed to the composition and structure of the materials used to fabricate the electrodes. Current research is focused on the development of electrode materials for wearable supercapacitors such as carbon nanotubes (CNT), graphene, transition metal oxides, conducting polymers, and their combined composites [251]. In the following, we report and discuss primary literature research relating to the benefits and limits of these materials (Table 1). Apart from textiles, these electrode materials can be inserted into plastics, sponge, and paper.

It is well-known that graphene composition has a single layer of hexagonal sp^2^ hybridized carbon atoms and possesses very high thermal and electrical conductivity, great mechanical and chemical stability, and high surface area (2630 m^2^∙g^−1^). These properties make graphene a suitable electrode material for wearable supercapacitors. Aboutalebi et al. synthesized at a large scale highly porous graphene oxide (GO) and reduced graphene oxide (rGO) fibers [252]. These fibers show a robust nature, electrical conductivity of 2508 ± 632 S∙m^−1^, and remarkably high specific surface area values (2605 and 2210 m^2^∙g^−1^ before and after reduction, respectively). The charge storage capacity is reported to be 409 F∙g^−1^, corresponding to 1 A∙g^−1^ with a rated capacity of 56 F∙g^−1^ for 100 A∙g^−1^, without losing its flexible nature. Niu et al. [253] fabricated an ultrathin supercapacitor using an ultrathin graphene electrode interlinked by polyethyleneterephthalate (PET) substrate. They used Au as the current collector and polyvinylalcohol or orthophosphoric acid as the electrolyte. The electrolyte utilizes the surface area of graphene more effectively in this supercapacitor thanks to the short diffusion lengths due to the electrolyte infiltration in the graphene layers. The specific capacitance of this ultrathin wearable supercapacitor is reported to be 285 F∙g^−1^, which is more than thrice the one reported for a conventional supercapacitor in the same experimental conditions (86 F∙g^−1^). The charge–discharge curve showed great Coulombic efficiency at 98%.

However, a drawback in the use of graphene nanosheets is the high attraction among the nanosheets, which strongly limits the utilization of the graphene large surface area.

To overcome this stacking problem, porous 3D graphene with high electrical conductivity, good chemical stability, and high open surface area has been also studied. Meng et al.synthesized graphene core-sheath fiber wrapped with 3D graphene sheet [254] resulting in great electrical conductivity with a highly exposed surface area. This designed fiber was found to be flexible and compressible and can be used as a textile for wearable supercapacitors. Even in the cycling bending, the specific capacitance of 30~40 μF∙g^−1^ was achievable.

Carbon nanotubes (CNTs) are well-known to be cylindrical molecules when acarbon atom is inasp^2^ hybridized state and appear like a rolled-up single layer carbon sheet (graphene). CNTs can be single-walled with a diameter less than 1 nm or they can multi-walled with diameters even greater than 100 nm. Their shape provides them chemical stability and high thermal and electrical conductivity. These properties make them suitable for applications in numerous fields. The use of carbon nanotubes in EDLC supercapacitors is due to their surprisingly high electrical conductivity (105 S∙cm^−1^) and surface area (2200 m^2^∙g^−1^). Highly flexible single-walled carbon nanotubes (SWCNTs) have been prepared by Niu et al. [255] for application in wearable supercapacitors. These buckled SWCNT films were deposited on polydimethylsiloxane (PDMS) substrate with enriched pre-strain. The capacity of surviving during strain is dependent on the wavelength of the SWNT buckled structure. The PDMS substrate will give a lower wavelength of buckled SWNT. They increase the pre-strain on the substrate PDMS, decreasing the wavelength of buckled SWCNT electrode. The buckling wavelength was 1.1 µm. This electrode, using H_2_SO_4_ as the electrolyte and PVA as the separator, gives unchanged capacitance even at 120% strain.

In wearable electronics, the electrode materials of supercapacitors can be damaged by bending or stretching, thus, the materials selected for the energy storage device should be robust. Self-healing materials can provide a solution to this problem because they are able to auto repair their internal and external damages. Wang et al. fabricated an electrical and mechanical self-healing wearable electrochemical capacitor from SWCNTs on a self-healing substrate. These self-healing materials are supramolecular links that support the TiO_2_ nanostructures. Their lateral movement causes the electrode materials to have contact, thus, repairing the damage by itself. The self-healing capacity of this electrode material increases by using a PVP-H_2_SO_4_ gel electrolyte. For this supercapacitor, the specific capacitance can be refurbished by 85%, even after the fifth cutting [256].

**Figure 10:**
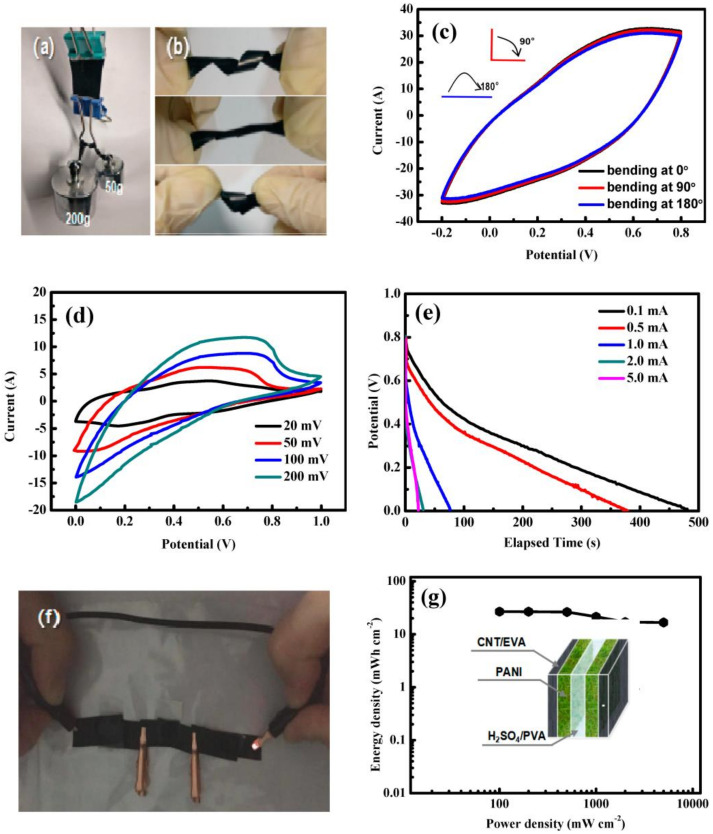
Digital photos of the PANI/CNT/EVA electrode: (**a**) mass loading, (**b**) twisting and binding, stretched and released. (**c**) Polarization curves of the PANI/CNT/EVA electrode under different bending angles. (**d**) Polarization curves and (**e**) discharge curves of solid-state symmetric supercapacitors at different scan rates and current densities. (**f**) Digital photo of the PANI/CNT/EVA flexible symmetric supercapacitors powering a LED. (**g**) Ragone plots PANI/CNT/EVA supercapacitor. Reproduced from reference [259] under CC BY 4.0 license.

Conducting polymers (CPs) have attracted the attention of scientists worldwide, owing to their high theoretical (100~140 mAh∙g^−1^), environmentally friendly, large voltage range, and large capacity/reversibility properties. These properties are also highly attractive for supercapacitors, and polyaniline (PANI), polypyrrole (PPy) and polythiophene (PTP) are the most used. Conducting polymers have lone pairs of electrons at oxygen and nitrogen available to electricity conduction, but their conducting state is strongly dependent on the polymer polarization condition (i.e., doped or undoped state), the dopant material [257], and to a smaller extent, on temperature, pH, ionic strength and solvent. Sunet al. [258] fabricated a smart supercapacitor which changes color depending on its working state. They deposited polyaniline (PANI) onto the carbon nanotube sheets, obtaining a specific capacitance of 308.4 Fg^−1^ and high stretchability and flexibility. The specific capacitance was well maintained even after 100% stretching for 200 cycles. A novel flexible electrode design made of a composite electrode of PANI/CNTs/ethylene-vinyl acetate copolymer (EVA) has been also proposed in reference [259]. EVA is a novel nontoxic plastic foam material with good buffering, shock, and chemical corrosion resistance and non-absorbent features. The results of this research have shown that the ion transmission capacity of EVA increases by the insertion of CNTs. In addition, PANI nanostructuring notably improves the specific surface area and capacitance of the flexible supercapacitor. High adhesion between active PANI and EVA/CNTs is easily obtained by the PANI electrodeposition process (Figure 10).

One of the main limitations of conducting polymers (CPs)-based electrode material is the performance instability under cycling due to volumetric swelling and contraction during polarization cycles. These effects are related to the polymer doping and de-doping with the surrounding ions, resulting in an important change in their conductivity status, but at the same time, they induce mechanical damages, and thus, fast capacitance decay to the supercapacitor. However, this issue can be solved by integrating CP hybrid EDLC/pseudocapacitors devices; for this reason, the existing literature considers CPs as one of the most promising electrode materials for flexible supercapacitors because of their high flexibility and simplicity in the synthesis processes [260].

Kim and co-workers reported graphene–polymer core-shell fiber (G-PF), which is developed using nitrogen and copper codoped graphene fiber cores coated with semiconducting polymers. This fabrication results in an electrical conductivity of 387.1 S∙cm^−1^ and a specific capacitance of 417.9 F∙cm^−3^ in G-PFs fiber [261].

Among all the metal oxide electrodes used in supercapacitors, MnO_2_ is widely used and acceptable due to its very high theoretical specific capacitance of 1400 F∙g^−1^. Yang et al. designed edge-oriented MoS_2_ thin film on Mo substrates. This electrode was flexible, with a high capacitance of 12.5 mF∙cm^−2^ [262]. Keeping in mind these results, Lu et al. invented a hybrid WO_3_-X/Au/MnO_2_ core-shell nanowire-based solid supercapacitor. This electrode was able to perform even under bending up to 180° angle without losing the electrochemical properties. This synthesized supercapacitor showed a specific capacitance of 1195 F∙g^−1^ for a current density of 0.75 A∙g^−1^ [263].

## 6. Outlook and Future Perspectives

In the development of wearable/flexible devices, the bottlenecks in material, such as scalable fabrication, low cost, low power consumption, and high stability, could be solved. As summarized in Table 2, high-quality soft materials have been proposed and tested in fuel cells, batteries, biofuel cells, and supercapacitors. Three main classes of materials (CNTs, graphene, polymer) are typically synthesized and assembled. Different composites and architectures (2D, 3D, foam, fibers) have been studied and fabricated according to different synthetic methods to produce highly efficient electrodes, electrolytes, and supports with outstanding electrochemical performances and good mechanical properties. Despite consistent literature work on soft materials for wearable energy devices being successfully done, the main limit at present is represented by the bending/twisting stability and the large scale production at sustainable costs.

In this review, we have proposed a deep understanding of outstanding literature in this topic, in order to provide a solid starting point for further advancements of wearable energy storage and conversion devices.

Notably, the key requirements for the transformation of traditionally bulky and weighty PEMFC into wearable, lightweight, and flexible devices are found in the material science aspects of current collectors and electrodes, with the proton-conducting membrane being thin and flexible itself. Metal nanostructures, conductive polymers, graphene sheets, and CNTs—often embedded in carbon-based fiber materials and/or polymer endplates—are actually the most investigated materials, as summarized in Table 2. While the selection of flexible and efficient electrode and electrolyte materials has been deeply studied, the simplification of the whole assembly is still a challenge. Soft materials—with high ohmic impedance for substrates—and hydrogen feeding—without heavy tanks—are under investigation.

Presently, supercapacitors or lithium-ion batteries have been utilized as wearable energy conversion devices, but they typically possess low energy density, undesirable safety, and low resistance to strong deformations. Their development is actually focused on these aspects. For example, self-healable materials are highly promising to solve this problem. Alternatively, Zn-based batteries can be utilized significantly with excellent flexibility, durability, and high energy density. With continuous improvements in design strategies and assembly technologies, several attempts have been made, but synergistic integration of high flexibility, safety, comfort, and high performance into flexible batteries remains an intense challenge.

Concerning wearable supercapacitors, graphene fiber and CNTs increase electrochemical properties and flexibility—suitable properties for wearable electronics’ energy storage. Conducting polymer electrodes have the advantage of eco-compatibility, but when they are used in wearable supercapacitors, the electrode damages itself on bending and stretching. Thus, conducting polymers should be synthesized using smart substrates that can repair the damage. The future prospect of wearable electronics is to explore new materials resources with good porosity for supercapacitor electrodes. In addition, environmentally friendly approaches should be kept in mind, which have been neglected until now.

Concerning biofuel cells, the batch of soft materials used actually is quite mature and consolidated, with the possibility to discover and introduce new and advanced conducting nanostructured copolymers—an emerging area in this topic—linked to the fabrication and decoration techniques used to functionalize the stretchable and foldable supports. To optimize the costs, due to the inherent lack of durability and degradability of the biological catalysts, more efficient immobilization procedures must be developed and the market price of these catalysts should be reduced, because one of the most limiting problems in biosensors and biofuel cells is still durability, long-term activity, and reusability. The achievement of higher and higher power densities is desirable, but it should not be the main aim. A useful application of these devices as wearable tools is linked to low-power electronics that can work continuously, but mainly intermittently; the use of capacitive and pseudocapacitive materials, such as anodes, is also an interesting possibility not extensively studied nowadays. For microbial fuel cells, miniaturization is the most urgent issue, especially for the very promising applications as biosensors for water quality, wastewater pollution load and potential remediation on-site. The future works on these biodevices should focus more on case studies, real deployments, and affordability of the technology in practical cases.

## 7. Conclusions

We have reported and discussed the evolution of research literature on soft materials for smart wearable fuel cells, batteries, biosensors, biofuel cells, and supercapacitors. Through a detailed analysis of the existing literature, we have demonstrated how excellent research efforts have been concentrated in the last ten years, covering the emergent topic of wearable devices. The observed general trends in material science applied to miniaturized, flexible, stretchable energy storage and conversion systems are to improve traditional electrode/electrolyte materials by fabrication of novelcomposites, by nanostructuring, by engineering novel architectures and by choosing the relevant combination of materials able to advance the performance of the whole device.

The extensive list of references of this review and the discussion provides a precious indicator of the numerous factors governing the growth of this topic and robustly contributes to the development of next-generation electrochemical energy conversion, storage, and biosensor wearable devices systems.

## Figures and Tables

**Table 1:** Comparison between the physicochemical properties of base materials used in the supercapacitor. Adapted from reference [252].

Physicochemical Properties	Carbon Materials	Metal Oxides	Conducting Polymers
Non-faradic capacitance	xxxx	xx	xx
Faradic capacitance	**o**	xxxx	xxxx
Conductivity	xxxx	x	xxxx
Energy density	x	xxx	xx
Power density	xxx	x	xx
Cost	xx	xxx	xx
Chemical stability	xxxx	x	xxx
Cycle life	xxxx	xx	xx
Easy fabrication	xx	x	xxx
Flexibility	xx	**o**	xxx

Very high: xxxx, high: xxx, medium xx, low x, very low o.

**Table 2:** Summary of soft materials for wearable energy conversion and storage devices.

Class of Soft Material	Electrodes	Electrolyte	Endplate/Current Collector/Support	Shape/ Conformation	Properties	Device	Ref.
Polymer	c	c	Au-deposited cyclo-olefin polymer	scotch-tape	flexible	PEMFC	[79]
Metal	Ag	c	Ag	nanowires	high electron conductive/flexible	PEMFC	[89,90]
CNTs	CNTs/carbon paper	c	plastic membrane	air-breathing cathode	lightweight, flexible, bendable, stretchable, twistable	PEMFC	[91]
Carbon-based	pyrolyzed - Zn/Co-ZIFs/polymer nanofibers	c	/	MOF fibers	ORR OER electrocatalyst	PEMFC, Zn–air batteries	[95]
polymer	c	c	Au-sputtered SOI wafers filled with Nafion	flat	thin, flexible	PEMFC	[97]
polymer	/	polyimide membrane	/	flat	lightweight, flexible ionic-electron conductive	PEMFC	[98]
polymer	/	in-situ gelled chitosan	/	flat	green, soft, highly proton conductive, flexible	PEMFC, DMFC	[99,100]
CNTs	CNT/Si and CNT/LMO composite yarn	c	c	fibers	flexible	LIBs	[120]
CNTs	MWCNTs/LiO composite yarns	c	absent	wire-shaped fiber	lightweight, flexible,	LIBs	[122]
CNTs	CNTs sheets/PDMS	c	c	sandwich structure	lightweight, flexible, stretchable	LIBs	[123]
metal nanostructures	hierarchical nanostructured 3D ZnCo_2_O_4_ arrays on carbon cloth	c	c	hierarchical	flexible and thin	LIBs	[126]
polymer	poly(vinylidene fluoride-co-hexafluoropropylene)-based gel electrolytes	c	c	flat	tensile strength, elasticity	LIBs	[134,135,136,137,138]
graphene	N-doped 3D porous graphene/cotton cloth	c	c	flat	high specific surface area, mechanical resistance, electron conductive	LIBs	[127]
polymer	c	poly(vinylidene fluoride-co-hexafluoropropylene) gel	c	flat	flexible, bendable	LIBs	[138]
polymer	c	polyurethane/poly(vinylidene fluoride) membrane	c	flat	stretchable ionic conductive	LIBs	[139]
CNTs Polymer Nanostructured metal	MnO_2_/SWCNTs cathode Zn foil anode	polyethylene oxide/TiO_2_ nanoparticles	carbon nanofiber mats current collector	flat	flexible	Zn–carbon	[145,146]
CNTs Nanostructured metal	3D CNTs/Ni foam cathode	c	c	flat	flexible	Zn–air battery	[150]
CNTs	Zn nanosheet/CNTs fibers anode ZnHCF/CNTs sheets cathode	c	c	coaxial-fiber	flexible, bendable, stable	Zn-ion battery	[154]
polymer	/	porous gelatin-based	/	flat	flexible, waterproof	Zn-ion battery	[156]
rGO/CNTs	rGO/CNTs/S	c	c	fiber	flexible	Li–S	[167]
graphene	graphene foam/S	c	c	cable-shaped	surface area, electron conductive, flexible	Li–S	[169]
graphene, CNTs	Ink filler	/	/	ink filler	flexible, electron conductive	EFCs	[213,214,215]
conductive polymer	polystyrene sulfonate/polyethyleneimine/polypyrrole	c	c	ink filler	flexible, electron conductive	EFCs	[216,217,218]
graphene, CNTs	paper anode Carbon sheets/CNTs/mediator complex cathode	sprayed selective separation layer	c		flexible, electron conductive	MFCs	[234,235]
GO, rGO	GO and rGO	/	/	fibers		SCs	[252]
graphene	graphene fiber wrapped with 3D graphene sheet	/	/	fiber wrapped 3D	flexible, electron conductive, compressible	SCs	[254]
graphene	thin graphene	c	Au current collector, PET substrate	thin	flexible, electron conductive,	SCs	[253]
CNTS	SWCNTs	PVP-H_2_SO_4_ gel	substrate		self-healing, flexible	SCs	[255]
polymer	PANI/CNTs/ethylene-vinyl acetate copolymer	c	c	nanostructured	flexible, bendable	SCs	[259]
transition oxides	Hybrid WO_3_-Au MnO_2_ -based	c	c	core-shell nanowires	flexible, bendable	SCs	[263]

“c”: conventional; “/”: not available.
